# Relationship Between Personality of Parents and Pediatric Post-Intensive Care Syndrome for a Family in the PICU: A Prospective, Observational Cohort Pilot Study

**DOI:** 10.3390/children12081056

**Published:** 2025-08-12

**Authors:** Misaki Kotani, Mitsuki Ikeda, Gen Aikawa, Hideaki Sakuramoto, Akira Ouchi, Haruhiko Hoshino, Keishun Boku, Yuki Enomoto, Nobutake Shimojo, Yoshiaki Inoue

**Affiliations:** 1 Department of Emergency and Critical Care Medicine, Faculty of Medicine, University of Tsukuba, Ibaraki 305-8575, Japan; 2 College of Nursing, Kanto Gakuin University, Yokohama 236-8501, Japan; 3 Division of Faculty Development, Nursing, Kindai University, Osaka 577-8502, Japan; 4 Department of Adult Health Nursing, College of Nursing, Ibaraki Christian University, Ibaraki 319-1221, Japan; 5 Adult Nursing (Acute Care) Department of Nursing, Faculty of Medical Technology, Teikyo University, Tokyo 192-0395, Japan; 6 Department of Pediatrics, University of Tsukuba Hospital, Ibaraki 305-8576, Japan

**Keywords:** Pediatric Intensive Care Unit, PICU, Post-Intensive Care Syndrome, family, anxiety, depression, PTSD, personality

## Abstract

**Introduction:** Post-Intensive Care Syndrome in Pediatrics (PICS-P) for families is a growing concern as receiving care in the Pediatric Intensive Care Unit (PICU) improves child survival. PICU parental stress may cause post-discharge psychiatric symptoms. Understanding personality-related distress is key for early intervention. This study examined whether parental personality traits correlate with such symptoms for PICS-P prevention. **Methods:** A cohort pilot study was conducted at a Japanese university hospital PICU (eight beds, 200–300 annual admissions, mandatory critical care consultation) between January and September 2022. Participants were parents of children admitted for longer than 1 week. Personality traits were investigated using the Big-Five-based test, and psychiatric symptoms were investigated using the Generalized Anxiety Disorder-7 (GAD-7), the Patient Health Questionnaire-9 (PHQ-9), and the PTSD Checklist-5 (PCL-5). The correlation between personality traits and psychiatric symptoms was investigated. **Results:** Among the 53 subjects who met the inclusion criteria, 25 gave consent to participate in this study. The correlation analysis revealed distinct patterns. Agreeableness demonstrated negative correlations: a moderately significant negative correlation with PTSD symptoms (*ρ* = −0.612, *p* < 0.05) and non-significant negative correlations with anxiety (*ρ* = −0.238) and depression (*ρ* = −0.060). Conversely, neuroticism exhibited positive correlations: a moderately significant positive correlation with anxiety symptoms (*ρ* = 0.539, *p* < 0.05), alongside non-significant positive correlations with depression (*ρ* = 0.318) and PTSD symptoms (*ρ* = 0.327). Regarding other personality traits, extraversion showed negative correlations with anxiety (*ρ* = −0.282), depression (*ρ* = −0.399), and PTSD (*ρ* = −0.438), conscientiousness displayed positive correlations with anxiety (*ρ* = 0.318), depression (*ρ* = 0.127), and PTSD (*ρ* = 0.467), while openness exhibited negative correlations with anxiety (*ρ* = −0.333), depression (*ρ* = −0.312), and PTSD (*ρ* = −0.309), although none of these associations were statistically significant. **Conclusions:** Lower levels of agreeableness and higher levels of neuroticism in parents are significantly associated with increased PTSD and anxiety symptoms, respectively, in the PICU setting. These personality traits may serve as predictors of parental psychological distress, suggesting their utility in informing targeted PICS-P interventions and preventative strategies.

## 1. Introduction

The first Pediatric Intensive Care Unit (PICU) for critically ill children was established in the U.S. in 1967 [1], and about 15 years later, Japan began to establish its own PICUs; there are currently 35 PICUs across Japan [2].

The PICU admits postoperative patients, including those undergoing highly invasive procedures such as for congenital heart disease, as well as patients with sudden hospital emergencies, emergency patients transported by ambulance, and critically ill patients transferred from other medical institutions. In recent years, interventions with medications and critical devices (such as ventilators and/or extracorporeal circulation) for critically ill pediatric patients have become more complex than in the adult field. As a result, establishing PICUs has become necessary to increase the life-saving rate of critically ill pediatric patients, as medical personnel need to possess the expert knowledge and experience to deal with such patients [3,4].

As the practice of pediatric intensive care has advanced, attention has increasingly turned to the emotional and psychological impact on patients and their families. In particular, individual differences such as parental personality traits may play a significant role in how families cope with the stress of a child’s critical illness. This is especially important in understanding Post-Intensive Care Syndrome in Pediatrics (PICS-P), a condition referring to the physical, cognitive, and psychological sequelae experienced by both pediatric patients and their families following intensive care. However, although PICS-P has been gaining recognition in recent years, it remains less well-known and less extensively studied than in the adult setting [5]. Considering the PICU experience and interactions with caregivers and family, there is less of a clear boundary between pediatric patients and their families in PICS-P from this perspective.

In the pediatric intensive care setting, families are more likely to experience intense stress than in the adult patient setting because their own child is in a life-threatening situation [6]. In fact, high rates of anxiety, depression, and PTSD symptoms have been reported in families after the child’s admission to the PICU, with 25.4%, 25.4%, and 34.3% of families experiencing anxiety, depression, and PTSD symptoms, respectively [7,8,9]. It has also been reported that these anxiety, depression, and PTSD symptoms do not resolve over time, but often become more pronounced and chronic even after discharge from the PICU [7,8].

The biopsychosocial (BPS) model emphasizes the role of psychological and sociological factors (such as thoughts, values, and daily life) in the development of psychiatric symptoms [10,11,12]. In intensive care, a comprehensive diagnosis should include biological, psychological, and sociological aspects [13]. Psychological care is essential not only for patients but also for the family, who are influenced by personality traits, resilience, and coping skills [14]. Studies show that families initially focus on the physical recovery of their child and later become aware of their own psychological burden [8]. Interaction with healthcare professionals during the PICU stay can help reduce their stress [15], making a continuous evaluation of psychiatric symptoms important.

There have been no studies investigating the relationship between the personality traits of parents of PICU patients and the development of psychiatric symptoms following discharge. However, under the framework of the BPS model, individual psychological traits and coping styles are considered important factors, and research from this perspective should be further promoted to better understand the psychological impact on families in the PICU setting. We hypothesized that such an investigation could elucidate the causes of psychological stress in patients’ parents and provide a steppingstone to early prevention and a reduction in psychiatric symptoms, which could lead to a reduction in PICS-P.

## 2. Materials and Methods

### 2.1. Setting and Patient Selection

This study was designed as a prospective observational study conducted at a Japanese university hospital with eight PICU beds and approximately 200–300 patients admitted annually. The PICU operates on a mandatory intensive care consultation model, providing postoperative care for both elective and emergency surgeries, as well as care for patients experiencing sudden clinical changes in other wards.

The participants in this study were parents of children who stayed in the PICU for at least one week between January and September 2022. This study also allowed participation by only one of the parents. The exclusion criteria included parents with a history of mental illness, the child’s death occurring during the study period, parents who were non-native speakers of Japanese, or children with no visits by either parent.

### 2.2. Data Collection

Demographic data on the affected children and their parents were collected from electronic medical records, and questionnaire data were collected from the participants. The gender, age, disease category, reason for admission, length of stay, and severity of illness (Pediatric Risk of Mortality III score: PRISM III [16]) of the patients and gender and age of parents were obtained from the electronic medical records.

Along with the consent form, a document containing a two-dimensional code linking to a questionnaire on psychological factors was distributed. After more than one week had passed since PICU admission and as the timing of discharge from the PICU approached, the feasibility of postal delivery was confirmed during a web-based meeting, and the documents were subsequently mailed. Participants were asked to provide an email address for receiving the consent form. If consent was obtained, a questionnaire assessing psychiatric symptoms was sent via email. This questionnaire was distributed at one month and three months following the child’s discharge from the PICU.

### 2.3. Measurements

A questionnaire evaluating both personality traits and psychiatric symptoms was administered to the participants. The personality traits were measured using the Japanese version of the Ten-Item Personality Inventory (TIPI-J) [17,18]. Psychiatric symptoms included anxiety (measured using the Generalized Anxiety Disorder-7 [GAD-7]) [19,20,21], depression (measured using the Patient Health Questionnaire-9 [PHQ-9]) [21,22,23], and post-traumatic stress disorder (PTSD; measured using the PTSD Checklist for DSM-5 [PCL-5]) [24]. These scales have each been shown to possess reliability and validity.

For anxiety, depression, and PTSD symptoms, cutoff values for each scale were used, and a symptom was considered to be present if the score was above the cutoff value. Cutoff values were determined using data from previous studies as follows: for anxiety, GAD-7 scores of 0 to 4 were defined as minimal, 5 to 9 as mild, 10 to 14 as moderate, and 15 to 21 as severe [19]. For depression, PHQ-9 scores of 0 to 4 were defined as minimal, 5 to 9 as mild, 10 to 14 as moderate, 15 to 19 as moderately severe, and 20 to 27 as severe [23]. The cutoff score for PTSD symptoms was 31 to 33 [24,25]. We chose 31 as the cutoff value to avoid overlooking patients.

### 2.4. Outcomes

The primary outcome was the correlation between personality traits and psychiatric symptoms. The secondary outcomes were the percentage of psychiatric symptoms and the difference between the father and the mother.

### 2.5. Statistical Analysis

Descriptive statistics were performed for each scale. Categorical variables were presented as numbers (%) and continuous variables as medians (interquartile range). Chi-square tests were performed for comparisons of categorical variables. For correlations, Spearman’s correlation coefficient was calculated. *p* < 0.05 was judged as statistically significant. IBM SPSS Statistics for Macintosh, Ver. 28.0.1.1 (IBM, Armonk, NY, USA) was used for statistical analysis.

For this pilot study, a pragmatic sample size of approximately 14 was determined to be feasible given resource constraints and the exploratory nature of this initial investigation into the relationship between parental personality and PICS-P. A post hoc sensitivity analysis revealed that for a sample size of n = 14, this study was powered to detect large correlation coefficients of approximately *ρ* = 0.6 or greater. While limited in detecting weaker correlations and generalizability, this sample size was deemed sufficient for the exploratory purposes in this pilot phase. In addition, as in previous studies, the Bonferroni correction was not applied in the current analysis; however, it will be considered in future full-scale studies.

## 3. Results

### 3.1. Selection of Participants

**Figure 1:**
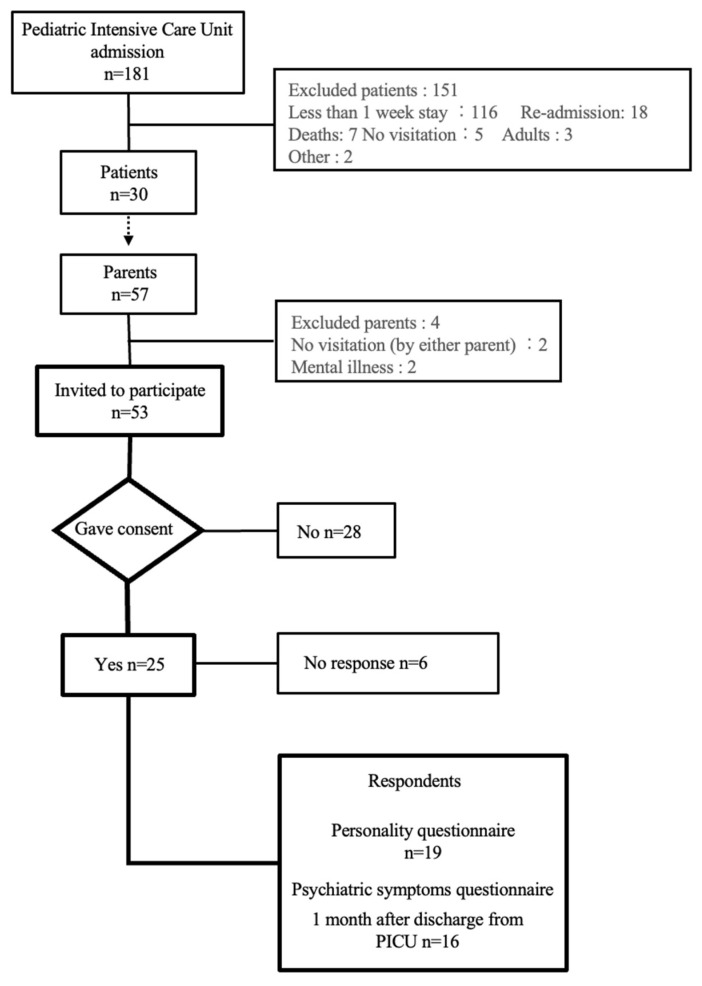
Flowchart of participant selection.

During the observation period, 181 patients were admitted to the PICU. Of these, 116 were admitted for less than a week, 18 were readmitted, 7 died, 5 had no visits, 3 were adult cases, and 2 were classified as “other”, resulting in the exclusion of a total of 151 patients. For the 30 children who were included, a total of 57 parents were considered for inclusion. After excluding 2 parents with mental illness and 2 parents who never visited from this study, 53 parents were invited to participate. Among these, 25 (47.2%) parents gave their consent to participate in this study (Figure 1). Among the 25 participants who provided informed consent, 6 did not complete the questionnaire and were therefore excluded from the analysis.

### 3.2. Parent Characteristics

In terms of parents’ characteristics, the median age was 32 for fathers (interquartile range (IQR): 31–35) and 33 for mothers (IQR: 31–35), with 15 (78.9%) of the parents being in their 30s.

With regard to personality, of the five components (extraversion, agreeableness, conscientiousness, neuroticism, and openness), fathers scored highest on the agreeableness component (median: 5.0; IQR: 4.5–5.5) and lowest on the extraversion component (median: 3.5; IQR: 3.0–5.5). Mothers scored highest on the agreeableness component (median: 5.5; IQR: 4.5–5.5) and lowest on the openness component (median: 3.5; IQR: 3.0–4.0). Overall, scores for the agreeable personality type were higher (median: 5; IQR: 4.5–5.5) (Table 1).

### 3.3. Patient Characteristics

The median age was 0 years (IQR: 0–2 years), and the largest number of patients (9; 53%) were infants under 1 year of age. Seven (41.2%) of the patients had cardiovascular disease (CVD), followed by four (23.5%) with respiratory disease and three (17.6%) with central nervous system disorders. Emergency admissions accounted for 13 (76.5%) patients, and 4 (23.5%) admissions were for surgery. The median length of stay was 14 days (IQR: 8–29 days), and about half of the patients (8; 47.1%) were admitted for a period between 10 and 30 days. The median PRISM III score for severity of illness was 18 (IQR: 11–20.25) (Table 2).

### 3.4. Psychiatric Symptoms One Month After Discharge

**Figure 2:**
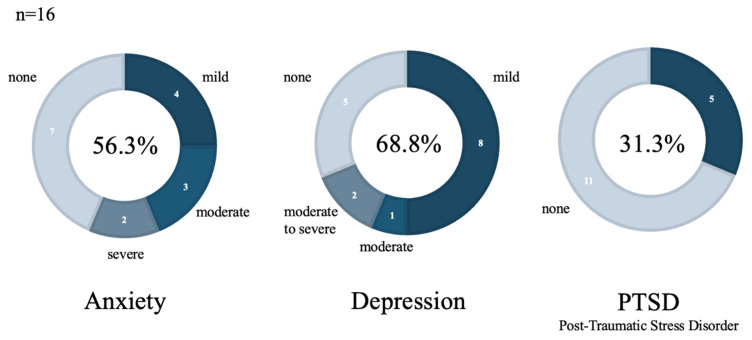
Psychiatric symptoms one month after discharge.

Among those who consented to participate in this study and whose children had been discharged for longer than one month, 16 (66.6%) parents completed the questionnaire at one month after discharge from the PICU. Of these, 9 (56.3%) had anxiety, 11 (68.8%) depression, and 5 (31.3%) PTSD symptoms at one month after discharge from the PICU (Figure 2).

**Figure 3:**
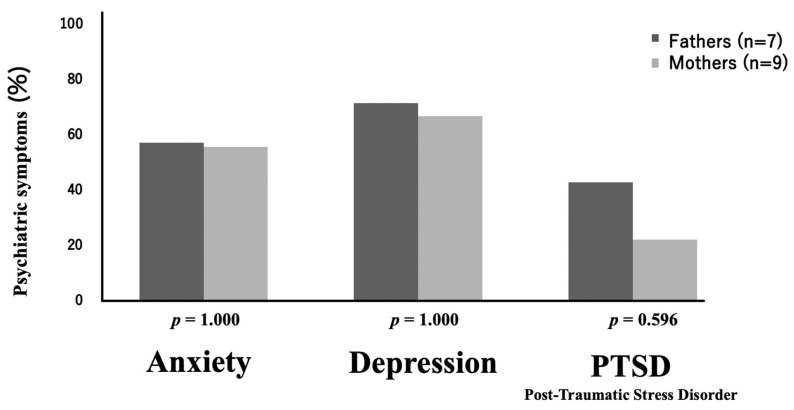
Percentages of psychiatric symptoms exhibited by fathers and mothers.

The percentages of psychiatric symptoms exhibited by fathers and mothers are shown in Figure 3. Anxiety was reported by four (57.1%) fathers and five (55.6%) mothers, indicating that there is no significant difference in the incidence of anxiety between fathers and mothers (*p* = 1.000). Likewise, depression was reported by five (71.4%) fathers and six (66.7%) mothers, demonstrating no significant difference in the incidence of depression between fathers and mothers (*p* = 1.000). Regarding PTSD symptoms, three (42.9%) fathers and two (22.2%) mothers had PTSD symptoms, with more PTSD symptoms occurring in fathers, although the difference was not statistically significant (*p* = 0.596).

### 3.5. Relationship Between Personality Traits and Psychiatric Symptoms at One Month After Discharge

**Figure 4:**
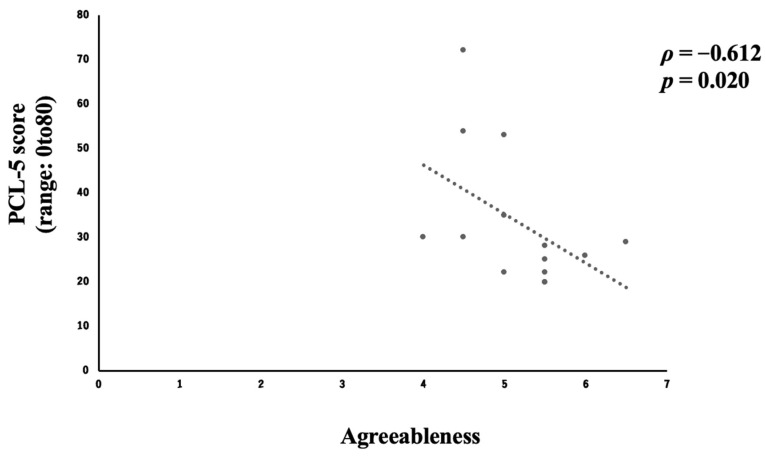
Correlation between agreeableness and PTSD symptoms.

**Figure 5:**
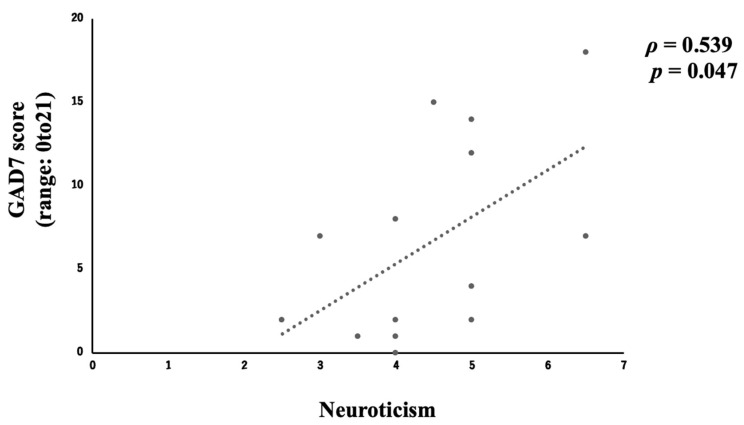
Correlation between neuroticism and anxiety.

Fourteen parents (58.3%) responded to both the personality and the psychiatric symptoms questionnaires at one month after discharge from the PICU. Analysis of personality based on the personality test revealed a moderate negative correlation (*ρ* = −0.612, *p* = 0.020) between agreeableness and PTSD symptoms (Figure 4) and a moderate positive correlation (*ρ* = 0.539, *p* = 0.047) between neuroticism and anxiety (Figure 5 and Table 3). Extraversion, conscientiousness, and openness were not significantly associated with anxiety, depression, or PTSD symptoms (*p* ≥ 0.05) (Supplementary Materials).

## 4. Discussion

This study investigated the association between personality traits and psychiatric symptoms in the families of patients admitted to the PICU. More than half of the families of patients admitted to the PICU had anxiety, depression, and PTSD symptoms at one month after discharge from the PICU, with no significant differences between fathers and mothers. The results of this study on the relationship between personality traits and psychiatric symptoms showed a moderate negative correlation between the agreeable personality type and PTSD symptoms at one month after discharge, and a moderate positive correlation between the neurotic personality type and anxiety at one month after discharge.

This pilot study (n = 14) provides preliminary insights, although its small sample size limits the statistical power and generalizability. Post hoc sensitivity analysis indicates that large correlations (*ρ* ≥ 0.6) were detected, even if weaker associations may have been missed. The findings should be interpreted as exploratory in nature, guiding future research with larger samples to confirm these initial trends and explore clinically relevant effect sizes. Furthermore, larger-scale studies are needed to comprehensively understand the complex relationship between parental personality and PICS-P.

A significant proportion of parents of patients admitted to the PICU experienced anxiety (56.3%), depression (68.8%), and PTSD symptoms (31.3%) at one month after discharge based on our study (Figure 3). In a previous study, anxiety, depression, and PTSD symptoms occurred at different rates (25.4%, 25.4%, and 34.3%, respectively) during admission, indicating a higher incidence of psychiatric symptoms in the present study [9]. One reason for this might be the small sample size in our study. However, similar to previous studies, the present study also showed that families experienced problems such as anxiety, depression, and PTSD symptoms after discharge from the PICU [7,8,9]. The results also suggest that classification of illness and urgency (such as elective surgery or emergency admission) may influence PICS-P [26]. In this study, emergency admissions accounted for the highest number of cases (76.5%), and severe CVD (such as postoperative congenital heart disease) accounted for about half the cases (41.2%). Although this may be characteristic of the facility where this study was conducted, we cannot exclude the possibility that these background factors influenced the results, as the proportion of patients with CVD was higher than that reported in previous studies [7,9]. Additionally, previous studies have conducted multi-center research on family satisfaction surveys, reporting that planned admissions have higher satisfaction levels than emergency admissions [27]. In this study, we focused solely on personality traits in our analysis; however, in the future, we aim to conduct a more detailed analysis that includes sociodemographic backgrounds and clinical factors as well.

In the evaluation of the relationship between personality traits and psychiatric symptoms, a moderate negative correlation was found between the agreeable personality type and PTSD symptoms (Figure 4), and a moderate positive correlation was found between a personality with neurotic tendencies and anxiety at one month after discharge (Figure 5). In other words, it can be concluded that PTSD symptoms are less likely to emerge in persons with a highly agreeable personality type, while anxiety symptoms are more likely to emerge in highly neurotic personality types.

Previous studies of hospitalized patients found a positive correlation between a personality with neurotic tendencies and PTSD symptoms, and a negative correlation between an agreeable personality type and PTSD symptoms [28,29]. In the present study, no significant correlation was found between neurotic tendencies and PTSD symptoms, although a negative significant correlation was found between agreeableness and PTSD symptoms, which is partially similar to the results of a previous study (Table 3) [29]. Parents with a highly agreeable personality type are thought to be less likely to develop PTSD symptoms because they have a high level of empathy, cooperate easily with those around them, tend to be supportive of others, and therefore have a supportive view of their severely ill children [30]. Regarding neuroticism and PTSD, previous studies have reported that the onset of psychiatric symptoms following discharge from the PICU tends to increase and become chronic over time [7,8]. These studies also suggest that while some families show a decrease in psychiatric symptoms over time, others experience an increase. In the present study, we conducted our analysis at one month after PICU discharge, and the results may differ with longer-term evaluations, such as at three months post-discharge. In future research, we aim to conduct comparative studies that take into account longer-term changes over time.

A previous study yielded similar findings, indicating that the neurotic personality type is positively correlated with anxiety [30]. On the other hand, parents with highly neurotic personalities are more likely to be depressed and emotionally unstable, which may lead to increased anxiety due to worry about their critically ill children and stress in the special environment of the PICU.

The results of this study suggest that personality traits may play a major role in the development of psychiatric symptoms in family members of patients admitted to the PICU. Screening family members upon admission to the PICU might help prevent the onset of psychiatric symptoms through proactive measures, including a consideration of intervention by a counseling psychologist early during admission. However, the specific methods of intervention should be further examined based on the results of future research. Although this study was focused on psychological aspects, the influence of sociological aspects such as family lifestyle cannot be ruled out, and further investigation is necessary.

### Limitations

As this is a pilot study, its generality might be limited. The first limitation of this study is the small sample size based on the theoretical power analysis. Since the analysis was conducted with a small sample size, it is possible that the results might differ when the sample size increases, and further research is needed. Additionally, some families in this study did not respond to the questionnaire after it was sent to them. It is necessary to analyze the data of those who did not respond and further examine the underlying factors, such as whether they could not find time in their daily lives to respond to the questionnaire, or whether they felt no need to respond due to a lack of psychiatric symptoms. In the future, we would like to conduct multi-center studies in order to secure a larger sample size.

Second, we excluded fatal cases and patients who were not expected to be able to leave the PICU in this study. Previous studies have reported no association between the severity of the illness affecting the child (PRISM III) and PTSD, although associations of PTSD symptoms with emergency hospitalization and the degree of anxiety caused by the possibility of the child’s death have been reported [26]. However, this aspect was hard to investigate because the subjects were excluded from this study from an ethical standpoint, taking into consideration the considerable psychological burden on families in situations where discharge from the PICU was not expected or the child was dying.

Third, although this study focused on personality traits, it is undeniable that background factors such as the classification of illness and urgency, as well as the presence or absence of a family history of mental illness and social background (as mentioned earlier), might have influenced the development of psychiatric symptoms. Investigations of these sociological aspects are also important, as previous studies have reported a higher psychiatric burden in the case of single parents and unemployed family members [7].

Furthermore, in this study, it was hard to assess personality characteristics before the PICU admission and at times of normalcy. Previous studies have reported that responses to certain questions are influenced not only by personality but also by other environmental and situational factors at the time of response [29]. The questionnaire regarding personality traits was completed after the children had physically recovered, which is presumably a time when the mental burden had been temporarily alleviated. However, one cannot deny the possibility that the children had already experienced a psychological burden and that the circumstances at the time of answering may have had some influence on the results. Therefore, in future research, we aim to include assessments conducted prior to hospitalization in order to establish baseline personality traits more accurately.

## 5. Conclusions

Our study of the relationship between psychological factors and psychiatric symptoms in the families of patients admitted to the PICU revealed that 56.3% of the families of patients admitted to the PICU had anxiety, 68.8% had depression, and 31.3% had PTSD symptoms at one month after discharge. This study revealed that personality traits may play a role in the development of psychiatric symptoms.

## Figures and Tables

**Table 1:** Data on parents’ characteristics.

Variable		Total	Father	Mother
**n (%)**		n = 19 (100)	7 (36.8)	12 (63.2)
**Age, median [IQR]** **Age group, n (%)**	20–29 30–39 40–49	32 [30.5–35.5] 2 (10.5) 15 (78.9) 2 (10.5)	32 [30.75–35.25]	32.5 [30.5–35]
Personality * (maximum score: 7), median [**IQR**]	Extraversion Agreeableness Conscientiousness Neuroticism Openness	4 [3.25–5] 5 [4.5–5.5] 4 [3–4.5] 4 [4–5] 3.5 [3–4.5]	3.5 [3.25–5.25] 5 [4.5–5.25] 4 [3.25–4] 4 [3.25–4] 4.5 [3–4.74]	4 [3.75–5] 5.5 [4.5–5.5] 4 [3–4.5] 5 [4–5] 3.5 [3–4]

* Note: Personality was assessed using the Ten-Item Personality Inventory—Japanese (TIPI-J). IQR: interquartile range.

**Table 2:** Data on patient characteristics.

Variable		n = 17
**Male, n (%))** **Female, n (%))**		8 (47.1) 9 (52.9)
**Age, n (%)**	<1 month <1 year old 1–5 years old 5–10 years old	2 (11.8) 7 (41.2) 6 (35.3) 2 (11.8)
**Disease, n (%)**	CVD CNS Respiratory Digestive system Other	7 (41.2) 3 (17.6) 4 (23.5) 1 (5.9) 2 (12.5)
**Admission category, n (%)**	Elective surgery Emergency admission	4 (23.5) 13 (76.5)
**Days in PICU, n (%)**	7–10 days 10–30 days 30–60 days 60–100 days >100 days	5 (29.4) 8 (47.1) 2 (11.8) 1 (5.9) 1 (5.9)
PRISM Ⅲ score *, median [**IQR**]		18 [11–20.25]

* Note: The PRISM III (Pediatric Risk of Mortality III) score is used as an index of severity of the patient’s illness. Scores are given in the range of 0 to 71, with higher scores indicating greater severity. CVD: cardiovascular disease; CNS: central nervous system.

**Table 3:** Correlations between personality traits and psychiatric symptoms.

Personality Traits	Anxiety n = 14	Depression n = 14	PTSD n = 14
**Extraversion**	−0.282	−0.399	−0.438
**Agreeableness**	−0.238	−0.060	−0.612 *
**Conscientiousness**	0.318	0.127	0.467
**Neuroticism**	0.539 *	0.318	0.327
**Openness**	−0.333	−0.312	−0.309

* *p* < 0.05.

## Data Availability

Due to the ongoing sub-analysis of the data collected for this study, the datasets are not currently publicly available. Access to the data may be granted by the corresponding author upon receipt of a justified request. Researchers seeking to utilize the data for subsequent investigations are invited to contact Ms. Misaki Kotani, the co-corresponding author, to discuss data availability.

## Supplementary Materials

The following supporting information can be downloaded at: https://www.mdpi.com/article/10.3390/children12081056/s1, Online Data Supplement : Correlations between personality traits and psychiatric symptoms.
