# Supplementary material for: Relationship Between Personality of Parents and Pediatric Post-Intensive Care Syndrome for a Family in the PICU: A Prospective, Observational Cohort Pilot Study
Source: Children (Basel). 2025 Aug 12;12(8):1056. doi: 10.3390/children12081056 (PMC12384791; doi:10.3390/children12081056)
Supplement: Supplementary file 1 [file children-12-01056-s001.zip › children-3732807-supplementary.pdf]

Online Data Supplement : Correlations between personality traits and psychiatric symptoms

Relationship Between Personality of Parents and Pediatric Post-Intensive Care Syndrome for a Family in the PICU: A Prospective, Observational Cohort Pilot Study

Misaki Kotani [1], Mitsuki Ikeda [1], Gen Aikawa [2], Hideaki Sakuramoto [3], Akira Ouchi [4], Haruhiko Hoshino [5], Keishun Boku [1], Yuki Enomoto [1,6], Nobutake Shimojo [1] and Yoshiaki Inoue [1,*]

[1] Department of Emergency and Critical Care Medicine, Faculty of Medicine, University of Tsukuba, Ibaraki 305-8575, Japan
[2] College of Nursing, Kanto Gakuin University, Yokohama 236-8501, Japan
[3] Division of Faculty Development, Nursing, Kindai University, Osaka 577-8502, Japan
[4] Department of Adult Health Nursing, College of Nursing, Ibaraki Christian University, Ibaraki 319-1221, Japan
[5] Adult Nursing (Acute Care) Department of Nursing, Faculty of Medical Technology, Teikyo University, Tokyo 192-0395, Japan
[6] Department of Pediatrics, University of Tsukuba Hospital, Ibaraki 305-8576, Japan

Running title: Relationship between Parent Personality and Pediatric Post-Intensive Care Syndrome

Running title: Relationship between Parent Personality and Pediatric Post-Intensive Care Syndrome

Address correspondence to:
Dr. Yoshiaki Inoue
Department of Emergency and Critical Care Medicine, Faculty of Medicine, University of Tsukuba, Tsukuba, Ibaraki, Japan.
E-mail: yinoue@md.tsukuba.ac.jp

Name and email of author:
Misaki Kotani (misakikotani812@gmail.com)

Although no statistically significant differences were found, a scatter plot illustrating a correlation between personality traits and psychiatric symptoms is presented here.

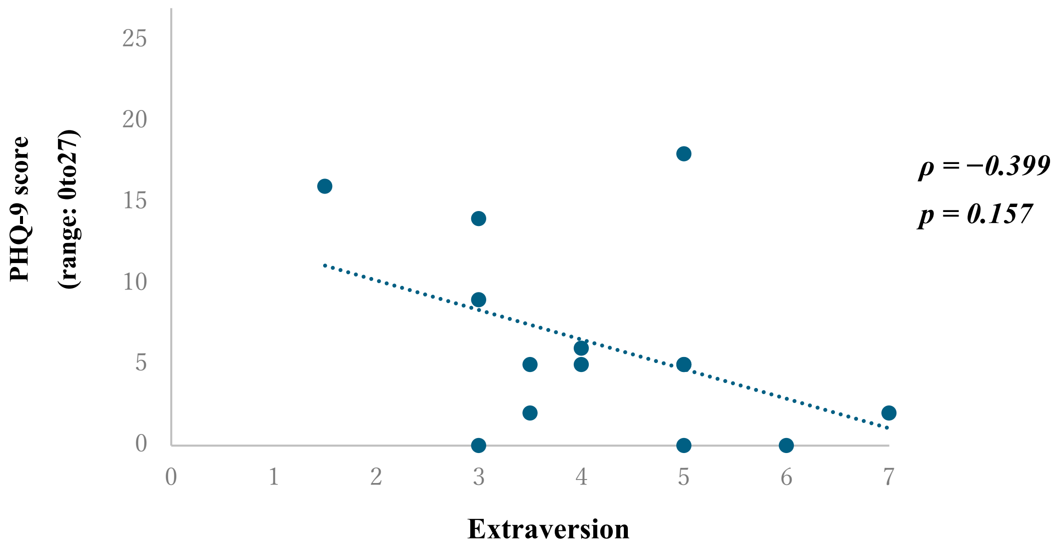

Figure 1: Correlation between extraversion and depression

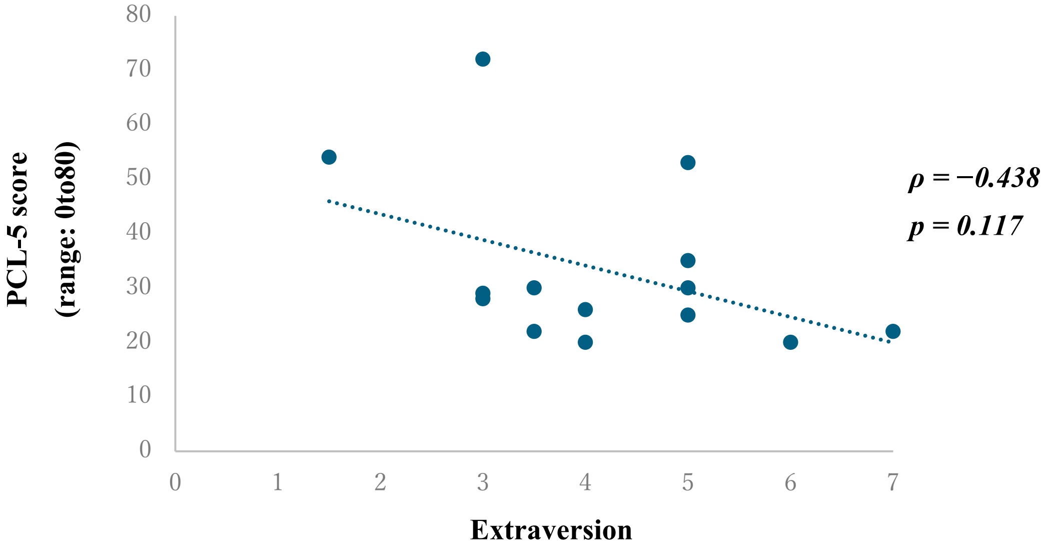

Figure 2: Correlation between extraversion and PTSD

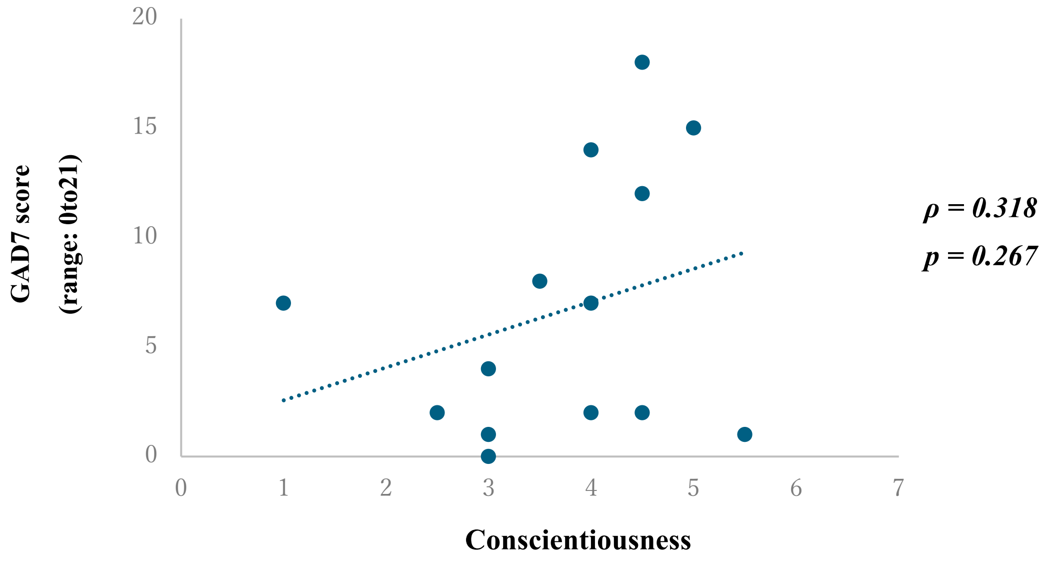

Figure 3: Correlation between conscientiousness and anxiety

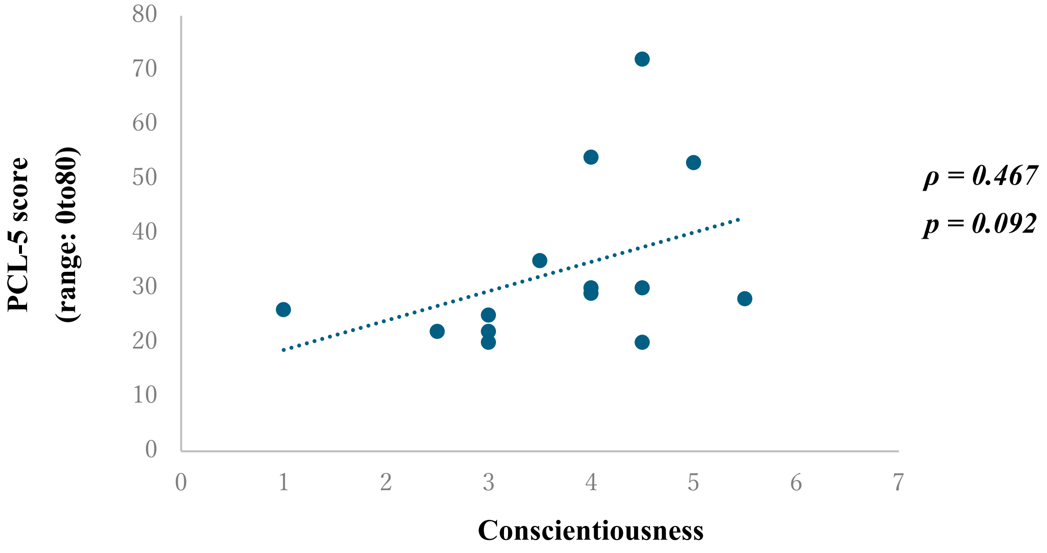

Figure 4: Correlation between conscientiousness and PTSD

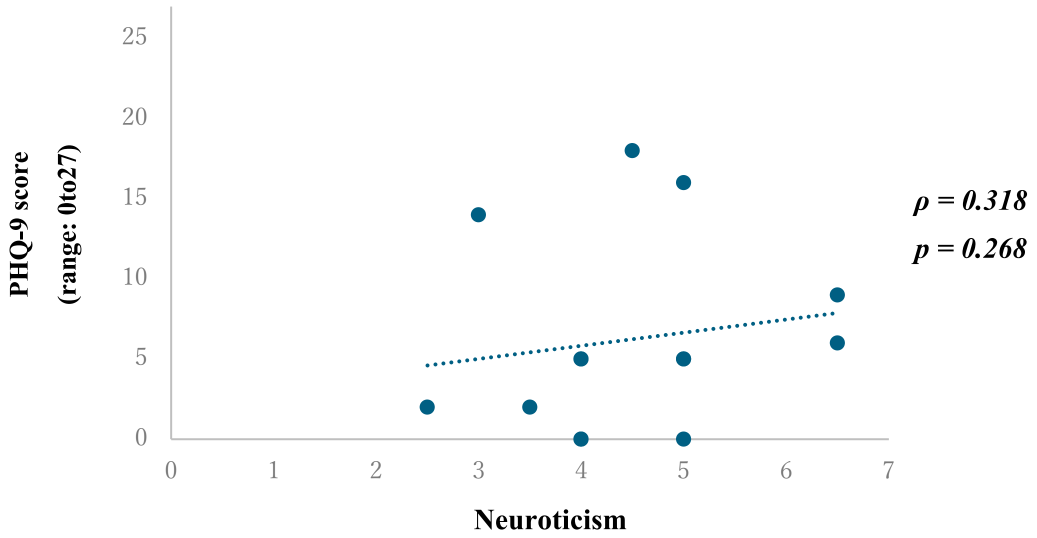

Figure 5: Correlation between neuroticism and depression

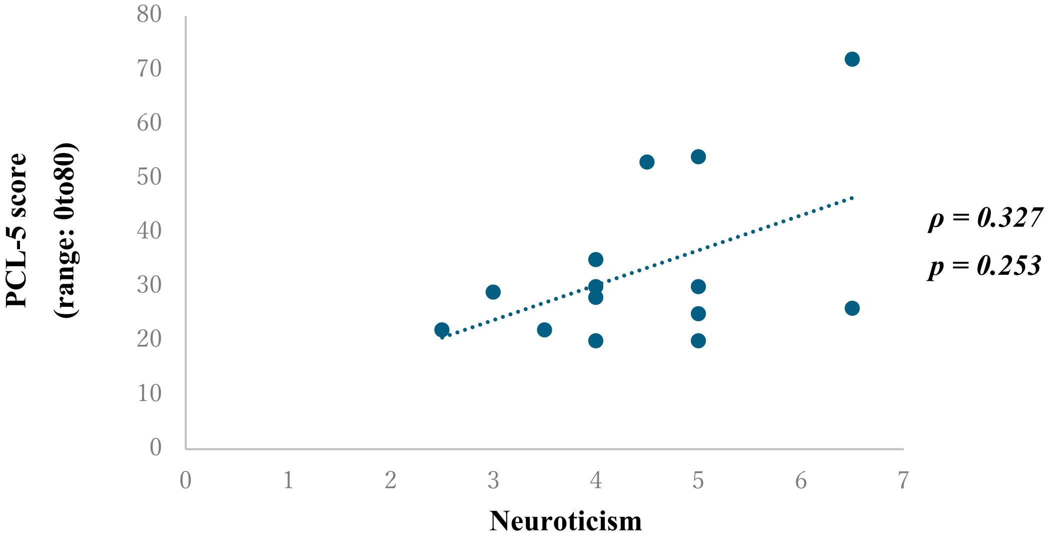

Figure 6: Correlation between neuroticism and PTSD

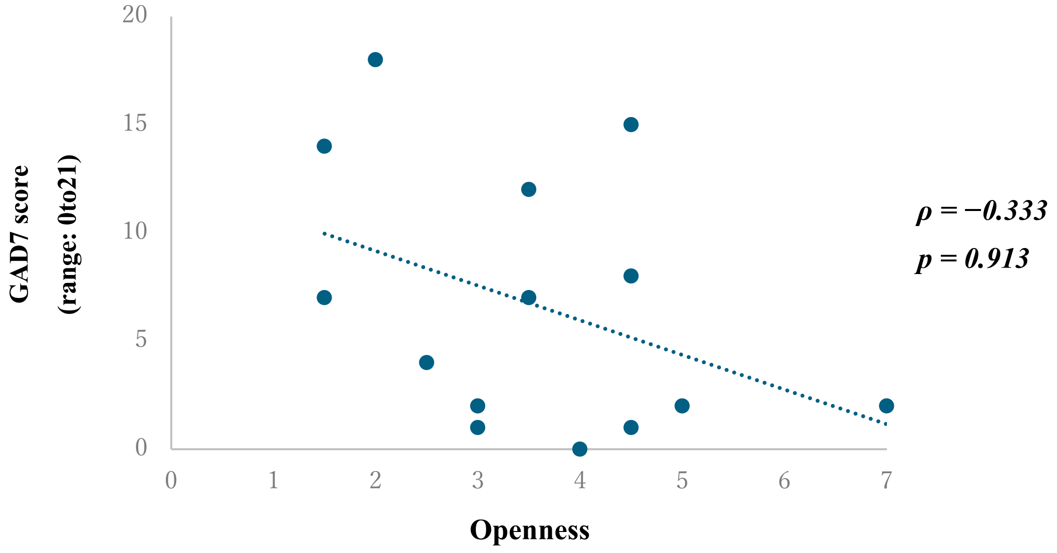

Figure 7: Correlation between openness and anxiety

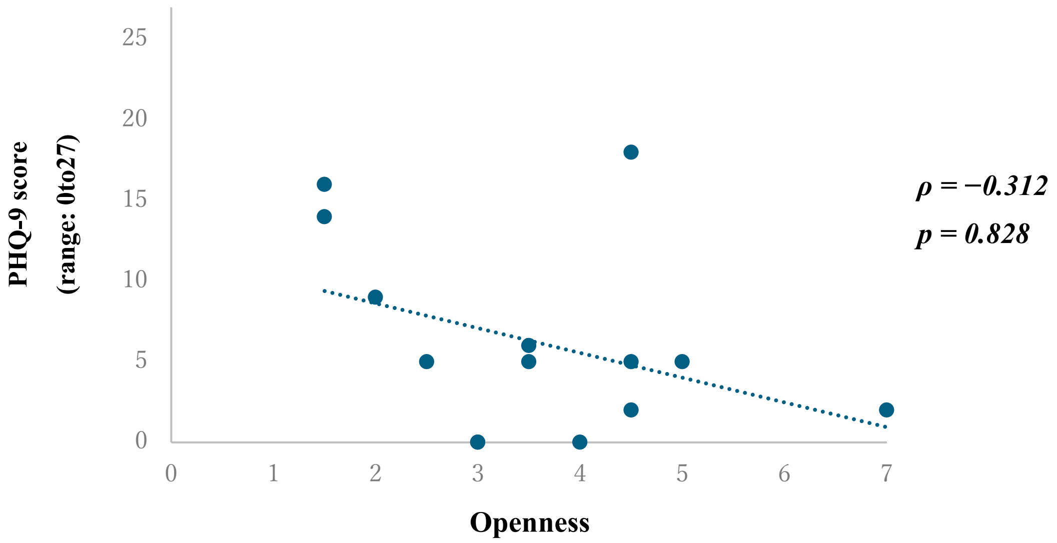

Figure 8: Correlation between openness and depression

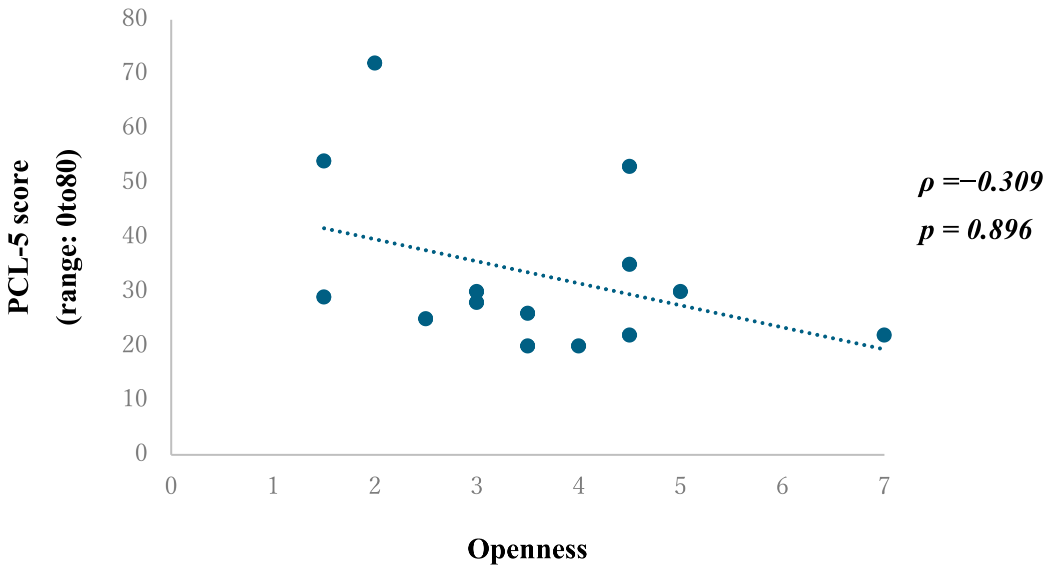

Figure 9: Correlation between openness and PTSD
